# Brainstem Respiratory Oscillators Develop Independently of Neuronal Migration Defects in the Wnt/PCP Mouse Mutant *looptail*

**DOI:** 10.1371/journal.pone.0031140

**Published:** 2012-02-17

**Authors:** Muriel Thoby-Brisson, Julien Bouvier, Derrick M. Glasco, Michelle E. Stewart, Charlotte Dean, Jennifer N. Murdoch, Jean Champagnat, Gilles Fortin, Anand Chandrasekhar

**Affiliations:** 1 UPR 3294 Neurobiology and Development, CNRS Institute of Neurobiology A. Fessard, Gif-sur-Yvette, France; 2 Division of Biological Sciences and Bond Life Sciences Center, University of Missouri, Columbia, Missouri, United States of America; 3 Mammalian Genetics Unit, Medical Research Council Harwell, Oxfordshire, United Kingdom; 4 Interdisciplinary Neuroscience Program, University of Missouri, Columbia, Missouri, United States of America; Institute of Neurology (Edinger-Institute), Germany

## Abstract

The proper development and maturation of neuronal circuits require precise migration of component neurons from their birthplace (germinal zone) to their final positions. Little is known about the effects of aberrant neuronal position on the functioning of organized neuronal groups, especially in mammals. Here, we investigated the formation and properties of brainstem respiratory neurons in *looptail* (*Lp*) mutant mice in which facial motor neurons closely apposed to some respiratory neurons fail to migrate due to loss of function of the Wnt/Planar Cell Polarity (PCP) protein Vangl2. Using calcium imaging and immunostaining on embryonic hindbrain preparations, we found that respiratory neurons constituting the embryonic parafacial oscillator (e-pF) settled at the ventral surface of the medulla in *Vangl2^Lp/+^* and *Vangl2^Lp/Lp^* embryos despite the failure of tangential migration of its normally adjacent facial motor nucleus. Anatomically, the e-pF neurons were displaced medially in *Lp/+* embryos and rostro-medially *Lp/Lp* embryos. Pharmacological treatments showed that the e-pF oscillator exhibited characteristic network properties in both *Lp/+* and *Lp/Lp* embryos. Furthermore, using hindbrain slices, we found that the other respiratory oscillator, the preBötzinger complex, was also anatomically and functionally established in *Lp* mutants. Importantly, the displaced e-pF oscillator established functional connections with the preBötC oscillator in *Lp*/+ mutants. Our data highlight the robustness of the developmental processes that assemble the neuronal networks mediating an essential physiological function.

## Introduction

The development of functional neuronal circuits requires appropriate migration of neurons from the germinal zone where they are born to their final position in the nervous tissue. Abnormal neuronal migration during development can cause neurological and cognitive impairments varying between mild to severe deficits [1]–[3]. Breathing is a spontaneous rhythmic behavior critical for life. However, mechanisms underlying the migration of neurons generating respiratory rhythm, and the consequences of their abnormal migration, have not been examined.

Respiratory rhythmogenesis relies on the activity of a brainstem respiratory rhythm generator located in the ventral medulla, and composed of two interacting oscillators: the preBötzinger complex (preBötC) that drives inspiration [4] and the parafacial respiratory group (pFRG) controlling pre-inspiratory and expiratory activities [5], [6]. It has been shown recently in rodents that respiratory oscillators emerge sequentially during development. At embryonic day (E) 14.5 in the mouse, rhythmic activity and chemosensitivity can be detected in the embryonic parafacial oscillator (e-pF) [7], while the preBötC oscillator activity appears one day later at E15.5 [8].

The final position of the e-pF is, ventrally, adjacent to the pial surface, and, dorsally, at the lateral edge of the facial motor nucleus (FMN) [7], [9], [10]. The e-pF and facial branchiomotor (FBM) neuron progenitors share expression of the visceral marker *Phox2b* [7], [9], although they are located in completely different domains of the hindbrain ventricular zone, along both the anterior-posterior axis (in *Egr2*-positive and -negative rhombomeres, respectively) and the dorsal-ventral axis (in dB2 and vMN domains, respectively). After exiting the cell cycle, migrating e-pF and FBM neurons become adjacent at E11.5 [9]. Subsequently, the e-pF neurons migrate radially around the FMN to reach the ventral (pial) surface, a physiologically important location close to relevant chemosensory signals and cerebral vascularization in the adult [11], [12]. Mechanisms underlying the migration of e-pF neurons are largely unknown. It is also not known whether defective e-pF migration/position can affect respiratory rhythm.

Here, we address these issues by testing whether a mutation that affects FBM neuron migration also affects e-pF neuron migration, and whether defects in e-pF positioning alter its respiratory-related functions. We used calcium imaging and pharmacological approaches to examine the functional and anatomical characteristics of the respiratory neuronal network in a mouse mutant where FBM neuron migration is abnormal. The transmembrane protein Van gogh-like 2 (Vangl2) is a component of the noncanonical Wnt/Planar Cell Polarity (PCP) signaling pathway [13], [14] required for tangential migration of FBM neurons from rhombomere 4 (r4) to r5-r7 in the hindbrain [13], [15], [16]. In *looptail* (*Lp*) mutant (*Vangl2−/−*) mice, we found that the e-pF oscillator was rostro-medially displaced, but could still be detected at the ventral medullary surface, was bilaterally synchronized, and exhibited characteristic functional properties. The location and properties of the preBötC oscillator were unaffected in *Lp* mutants. Thus, hindbrain respiratory oscillators develop and establish function independently of mechanisms regulating FBM neuron position.

## Methods

### Ethics Statement

Animal maintenance and experiments were performed in accordance with French National (JO 87-848) and European (86/609/CEE) legislation on animal experimentation, and following the guidelines of the Animals (Scientific Procedures) Act 1986 of the UK Government and the University of Missouri Animal Care and Use Committee (Animal Welfare Assurance Number A3394-01). The institutional animal care committees at CNRS, Gif-sur-Yvette and the University of Missouri specifically approved this study.

### Animals

The *looptail* (Lpt/Le) inbred strain, which carries the *Vangl2^Lp^* mutation, was originally obtained from the Jackson Laboratory (Bar Harbor, Maine, USA). The colony was maintained by brother-sister mating for over 100 generations, then bred to congenicity on the C3H/HeH background. Heterozygous animals were intercrossed to generate litters containing *+/+, Lp/+* and *Lp/Lp* embryos, and mice were genotyped by pyrosequencing for the *Vangl2* mutation as described previously [17]. The *Lp* mutation generates a potentially non-functional Vangl2 protein that localizes poorly to the plasma membrane [18], [19].

Mice (*Lp/+*) were mated overnight; the day of finding the vaginal plug was considered as embryonic day (E) 0.5. Pregnant dams were shipped before E14.5 from MRC Harwell to CNRS, Gif-sur-Yvette in accordance with UK Home Office and EU guidelines. The size and gross anatomy of all embryos obtained from *Lp*/+ incrosses, as well as the spontaneous activity recorded from whole hindbrain preparations of all three genotypes (+/+, *Lp*/+, *Lp*/*Lp*) revealed that embryonic development in these litters (C3H/HEH background) was delayed by about one day compared to standard staging used in previous studies (C57BL6/DBA2 and OF1 backgrounds) [7]. Consequently, the e-pF, which emerges at E14.5 [7], was actually examined at E15.5 for +/+, *Lp*/+ and *Lp*/*Lp* embryos in the present study. Similarly, the pre-Bötzinger complex, which emerges one day later at E15.5 [8], was examined at E16.5 for all embryos. Moreover, since the *looptail* mutation is lethal at birth, it was not possible to further test the respiratory function at post-natal stages, and all experiments were performed at embryonic stages.

### In vitro preparations

**Figure 1:**
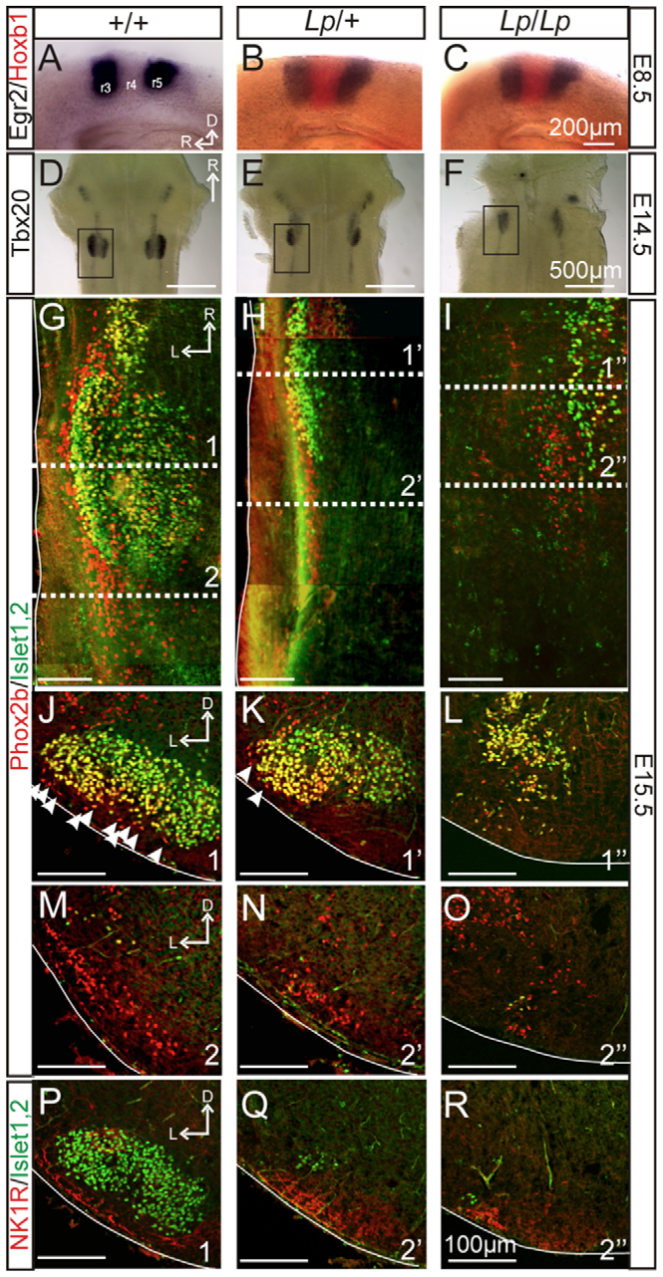
Location of the facial motor nucleus and Phox2b-positive cells in the hindbrain of *Lp* mutant embryos. A–C: In situ hybridization of *Egr2* (black) and *Hoxb1* (red) in E8.5 whole hindbrain preparations (lateral view) obtained from a *+/+* (A), *Lp/+* (B) and *Lp/Lp* (C) embryos. Wt (+/+) embryo processed for *Egr2* in situ only. Expression of *Egr2* in rhombomere 3 (r3) and r5, and of *Hoxb1* in r4 are unaffected in *Lp*/+ and *Lp/Lp* embryos. D–F: In situ hybridization of *Tbx20* expression in E14.5 whole hindbrain preparations (ventral view) obtained from +/+ (D), *Lp/+* (E) and *Lp/Lp* (F) embryos. Facial motoneurons failed to migrate properly both in *Lp/+* and *Lp/Lp* embryos. The black squares indicate the regions illustrated in G, H and I. G–O: Anti-Phox2b (red) and anti-Islet1,2 (green) immunofluorescence on whole hindbrain preparations (G–I, ventral view) and transverse sections (J–O) through E15.5 hindbrains of *+/+* (G, J, M), *Lp/+* (H, K, N) and *Lp/Lp* (I, L, O) embryos. White arrowheads in J and K point to Phox2b-positive cells located ventral to the facial nucleus. Numbered dashed lines in G–I refer for each genotype to the axial level corresponding to the sections illustrated in J–R. P–R: Anti-NK1R (red) and anti-Islet1,2 (green) immunofluorescence on transverse sections through E15.5 hindbrain preparations obtained at level 1 for a *+/+* embryo (P), and at level 2 for *Lp/+* (Q) and *Lp/Lp* (R) embryos. Slices illustrated in M and P, N and Q, O and R, correspond to adjacent slices obtained from the same animal, thus showing possible co-expression of several markers in the same neuronal population. Immunostainings indicate the presence of Phox2b-positive or NK1R-positive cell groups present at the ventral surface of the hindbrain for all genotypes. D: dorsal, L: lateral, R: rostral.

**Figure 2:**
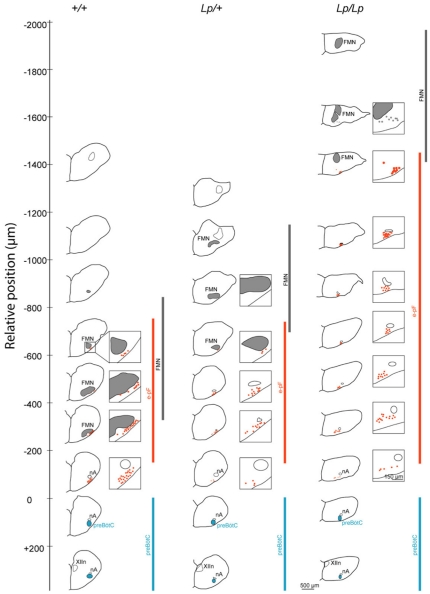
Rostro-caudal distribution of facial motor neurons and respiratory neurons in the hindbrain of wild-type and *Lp* mutant embryos. Half slice drawings were obtained from images of brainstem slices labeled with anti-NK1R and Islet1,2 antibodies (dorsal up). The red dots indicate individual e-pF neurons, the gray areas indicate the FMN and the blue ovals indicate the preBötC. The rostro-caudal extents of the preBötC (blue), the e-pF (red) and the FMN (gray) are indicated by vertical colored bars. The numbers on the left of each drawing indicate the rostro-caudal position (in µm) of the slice relative to the preBötC position, which is defined as zero (see the full scale on the left of +/+). Note that the e-pF is more rostrally and loosely distributed in the *Lp/Lp* preparation compared to the +/+ and *Lp/+* preparations. Similar distributions were observed in a second preparation for each genotype. XIIn: hypoglossal nucleus, nA: nucleus ambiguus, FMN: facial motor nucleus.

Pregnant females were sacrificed by cervical dislocation at the 14^th^, 15^th^ or 16^th^ day of gestation (E14.5, E15.5 or E16.5). Uterine horns were removed from the mother and embryos were excised from their individual bag and kept until the recording session in oxygenated artificial cerebrospinal fluid (a-CSF) at 25°C. The a-CSF composition (in mM) was: 120 NaCl, 8 KCl, 1.26 CaCl_2_, 1.5 MgCl_2_, 21 NaHCO_3_, 0.5 Na_2_HPO_4_, 30 glucose, pH 7.4. To induce acidification the pH of the a-CSF was lowered to 7.2 by decreasing the NaHCO_3_ concentration to 10.5 mM while adjusting the NaCl concentration at 130.5 mM. We used two different preparations to examine the two respiratory oscillators: isolated brainstems for the e-pF and transverse slices for the preBötC. Brainstem and slice preparations were dissected in the a-CSF solution at 4°C and obtained as described previously [7], [8]. Briefly, a rostral section performed at the junction between the mesencephalon and the rhombencephalon and a caudal section performed below the fourth cervical roots allowed to isolate brainstems from the central nervous system (“whole hindbrain” preparation). After embedding brainstems in an agar block, transverse medullary slice preparations were obtained by serially sectioning the preparation in the transverse plane from rostral to caudal using a vibratome (Leica). A 450 µm thick slice isolating the preBötC oscillator was obtained with an anterior limit set 200–300 µm caudal to the posterior extremity of the facial motor nucleus (FMN). In the *Lp/+* heterozygote, we had to adapt this procedure due to the mislocation of the FMN. Based on immunostainings (Figs. 1 and 2), we determined that the FMN is 200–300 µm more rostral than in a wild-type embryo. We therefore took the slice that was 500–600 µm more caudal than the posterior extremity of the FMN. In the homozygous *Lp/Lp* mutant that exhibits a completely open neural tube, anatomical landmarks normally used to obtain the transverse slice isolating the preBötC, such as the position of the FMN, the presence of the inferior olive, and the outline of the fourth ventricle, could not be detected visually in transmitted light. In addition after isolation, the malformed hindbrain was extremely fragile and had to be very carefully manipulated. Therefore, slices containing the preBötC oscillator in *Lp/Lp* embryos were obtained as follows: we made consecutive 450 µm thick slices over the entire length of the open neural tube and tested activity for all of them. After isolation, the preparations (isolated brainstems (e-pF) or transverse slices (preBötC)) were first incubated in the calcium indicator before being transferred into the recording chamber where they were continuously superfused with oxygenated a-CSF and maintained at 30°C. To allow imaging of neuronal activities in the recording chamber, isolated preparations were placed ventral (pial) side up for brainstems (e-pF) and rostral side up for transverse slices (preBötC).

### Calcium imaging

Preparations were incubated for 40 min in oxygenated a-CSF containing the cell-permeable calcium indicator dye Calcium-Green 1AM (10 µM; Molecular Probes, Inc., Eugene, OR). After a 30 min recovery period in the recording chamber to wash out the excess dye and to allow spontaneous activities to emerge, an epifluorescent illumination system on an E-600-FN upright microscope (Nikon) equipped with a fluorescein filter block was used to excite the dye and capture the emitted light. Fluorescence images were captured with a cooled CCD camera (Coolsnap HQ, Photometrics, Tucson, AZ) using an exposure time of 100 ms in overlapping mode (simultaneous exposure and readout) during periods of 30 to 120 sec and analyzed using Metamorph software (Universal Imaging Corporation, West Chester, PA). The average of intensity in a region of interest was calculated for each frame. Mean baseline fluorescence was measured over a period between two spontaneous events and was used to normalize the ΔF signal which was then displayed as (ΔF/F). The distributions of active cells obtained in these experiments depict only cells that took up the dye, and which could be detected with the imaging parameters used.

### Pharmacological treatments

Drugs were obtained from Sigma (St Louis, MO, USA), dissolved in a-CSF and bath-applied for 5 to 10 minutes at the following concentrations: 0.1 µM Substance P (SP); 20 µM 6-cyano-7-nitroquinoxaline-2,3-dione (CNQX); 10 µM Riluzole (Ril). Frequency measurements were performed during the last three minutes of drug application. Frequency values are given as means ± SEM, and statistical significance was tested using a Student's *t* test or one-way ANOVA when appropriate. Differences were assumed to be statistically significant at p<0.05.

### Electrophysiology

Phrenic nerve activity in whole hindbrain preparations was recorded from the C4 roots using glass micropipette suction electrodes (100 µm tip diameter). The micropipette was filled with a-CSF and connected to a high-gain AC amplifier (7P511; Grass Instruments) through silver wires. The collected signals were filtered (bandwidth, 3 Hz to 3 kHz), rectified and integrated using an electronic filter with a time constant of 100 ms (Neurolog System), then stored on a computer via a digitizing interface (Digidata 1322A; Molecular Devices) and analyzed with the PClamp9 software (Molecular devices).

### Immunostaining

Antibody staining was performed on frozen sections or whole hindbrain preparations. Brainstem preparations were fixed for 2 to 3 hours in 4% paraformaldehyde. For frozen sections, tissues were cryoprotected in 30% sucrose-PBS (phosphate-saline buffer) overnight, embedded in Tissue Tek (Leica), and cryo-sectioned at 20 µm. Preparations (slices and whole hindbrains) were incubated for 30 minutes in 1% fetal bovine serum (FBS) and 0.5% Triton X-100, and incubated overnight at 4°C with primary antibodies: mouse anti-islet1,2 (1/250, DSHB, Iowa City, IA), a rabbit anti-NK1R (1/5000, Sigma) and a rabbit anti-Phox2b (1/1500; Gift from C. Goridis, ENS, Paris, France). After several rinses, preparations were incubated for 1 hr with secondary antibodies: FITC-conjugated goat anti-mouse (1/1000, Abcam) and Alexa Fluor 594-conjugated goat anti-rabbit IgG (1/400, Invitrogen). Stained preparations were coverslipped and mounted in Vectashield medium (Vector Labs) for preserving fluorescence. Slides were scanned on an SP2 confocal microscope (Leica Microsystems). A contrast enhancement and a noise reduction filter were applied to the images using Adobe Photoshop. Control experiments in which the primary antibodies were replaced by normal serum showed no labeling.

### *In Situ* Hybridization

Hindbrains were dissected from embryos, fixed overnight in 4% paraformaldehyde, and dehydrated in 100% MeOH before processing. Tissues were permeabilized with proteinase K, hybridized with digoxygenin-labeled *Tbx20* riboprobe, incubated with anti-digoxygenin alkaline phosphatase-conjugated antibodies, and developed with NBT/BCIP substrates (Roche). For two-color in situs, tissues were hybridized with digoxygenin-labeled *Egr2* and fluorescein-labeled *Hoxb1* probes, and first incubated with anti-digoxygenin alkaline phosphatase-conjugated antibody (NBT/BCIP substrate) to detect *Egr2* expression. After extensive washing, the tissues were incubated with anti-fluorescein alkaline phosphatase-conjugated antibody (FastRed substrate, Sigma) to detect *Hoxb1* expression. The hindbrains were cleared in glycerol and flat-mounted for photography. Images were acquired with an Olympus digital camera, and adjusted for brightness and contrast using Adobe Photoshop. *Tbx20* cDNA was obtained from Dr. Michele Studer (University of Nice Sophia Antipolis, Nice, France). *Egr2* and *Hoxb1* cDNAs were generated from total RNA using specific primers.

## Results

### Phox2b-positive/Islet1,2-negative e-pF neurons are mislocated in *looptail* (*Lp*) mutants

Given that e-pF neurons are located normally in close proximity to the FMN [7], [20], and that the FMN is misplaced in *Lp/+* and *Lp/Lp* embryos [15], [16], we used various e-pF molecular markers to examine its location in *Lp* mutants.

Since hindbrain morphology is largely disturbed in the *Lp* mutants due to the fully open neural tube, we first examined whether rhombomeric patterning in the hindbrain is preserved in *Lp* mutants. We performed wholemount in situ hybridization on E8.5 hindbrains with *Egr2* and *Hoxb1* probes, which are expressed in rhombomeres (r) 3 and r5 [21], and in r4 [22], respectively. *Egr2* and *Hoxb1* were expressed normally in *Lp*/+ and *Lp*/*Lp* hindbrains, indicating that rhombomere boundaries and identities were not affected (Fig. 1A–C).

Wholemount in situ hybridization for Tbx20, a T-box transcription factor expressed by migratory viscero- and branchio-motor neurons of the hindbrain [23], [24], was performed in E14.5 embryos, a stage at which the FMN has completely formed at the ventro-lateral (pial) surface of a brainstem area mostly derived from rhombomere 6 (r6; Fig. 1D). Although rhombomeres are not evident after E12.5, we will refer to positions of neurons in specific rhombomere-derived locations in older embryos based on the final positions of facial motor neurons in r6 in WT [25] and in r4 in *Lp*/*Lp* E12.5 embryos [16], and of glossopharyngeal and vagal motor neurons (nA) in r7/8 [26] in all genotypes. Accordingly, in *Lp/+* embryos (n = 6), FBM neurons formed a nucleus (FMN) more rostrally than in wild-type embryos, approximately r5 (Fig. 1E). Moreover, in *Lp/Lp* embryos (n = 6), FBM neurons failed to move caudally, but migrated laterally within r4 to form an ectopic nucleus in the dorsal part of the hindbrain (Fig. 1F; see also Figs. 1L and 2) [16].

We next examined whether the distribution of e-pF neurons was spatially correlated with the position of the FMN in the different genotypes. The facial motor neurons and e-pF neurons can be discriminated by their different immunoreactivity against Islet1,2 (a marker for motor neurons) and NK1R (a marker for e-pF neurons), while they both express Phox2b [9], [27]. In E15.5 wild-type embryos (n = 2 wholemount and 2 sectioned), Phox2b^+^/Islet1,2^−^ neurons were distributed lateral to the FMN, and extending caudally and ventrally (Fig. 1G, J, M) [7]. NK1R immunostaining revealed a similar distribution (Fig. 1P) [7]. In *Lp/+* embryos (n = 4 wholemount and 2 sectioned), Phox2b^+^/Islet1,2^−^ cells were located at the ventral surface of the hindbrain where they formed a column extending from the caudal end of the mis-located FMN (in r5) up to 300–400 microns more caudally (Fig. 1H), but not lateral to the FMN. This slightly medially-displaced distribution of Phox2b^+^/Islet^−^ neurons was also evident in transverse sections at various rostro-caudal levels using either Phox2b (Fig. 1K, N) or NK1R immunostaining (Fig. 1Q). In *Lp/Lp* embryos (n = 3 wholemount and 2 sectioned), only a few motor neurons could be detected at the ventral surface of the hindbrain in a r4-like position (Fig. 1I), consistent with the rostral and dorsal location of FBM neurons seen in *Tbx20* in situs. Importantly, Phox2b^+^/Islet1,2^−^ cells were still found near the pial surface, but at a more rostral position than in +/+ and *Lp/+* embryos. In transverse sections at the axial level where motor neurons were present (Fig. 1I, section 1), Phox2b^+^/Islet^−^ cells were absent from the ventral (pial) surface (Fig. 1L). More caudally (Fig. 1I, section 2), Phox2b^+^/Islet^−^ cells were detected at the surface of the hindbrain where they also expressed NK1R (Fig. 1O, R). These data demonstrate that in *Lp/+* and *Lp/Lp* embryos, the abnormal location of the FMN does not impact the ability of Phox2b^+^/Islet^−^/NK1R^+^ e-pF-like neurons to reach the ventral medullary surface, but at dorsal and medial positions compared to wild-type embryos.

Using immunostainings performed on transverse slices covering the entire rostro-caudal extension of the hindbrain, we constructed a map of the positions of the e-pF-like and FBM neurons for each genotype, as shown on Figure 2. The nucleus ambiguous (nA) served as a reference for rostro-caudal level. We found that the position of the preBötzinger complex (preBötC), relative to the nA, was not affected by the mutation. Therefore, the e-pF-like and FBM neurons were positioned relative to the location of the nA and the preBötC. This atlas clearly indicates that 1) the FMN is rostrally displaced in *Lp/+* and *Lp/Lp* embryos, 2) the e-pF lays caudally but not laterally to the misplaced FMN in the mutants, and 3) in *Lp/+* embryos, the e-pF is found in a similar rostro-caudal position to that of +/+ embryos, whereas it is rostrally displaced and more dispersed in *Lp/Lp* mutants. Altogether, these data suggest that the *looptail* mutation affects the caudal, but not the ventral, migration of e-pF-like neurons.

### Rhythmically active neurons are present at the ventral surface of the hindbrain in *looptail* mutants

**Figure 3:**
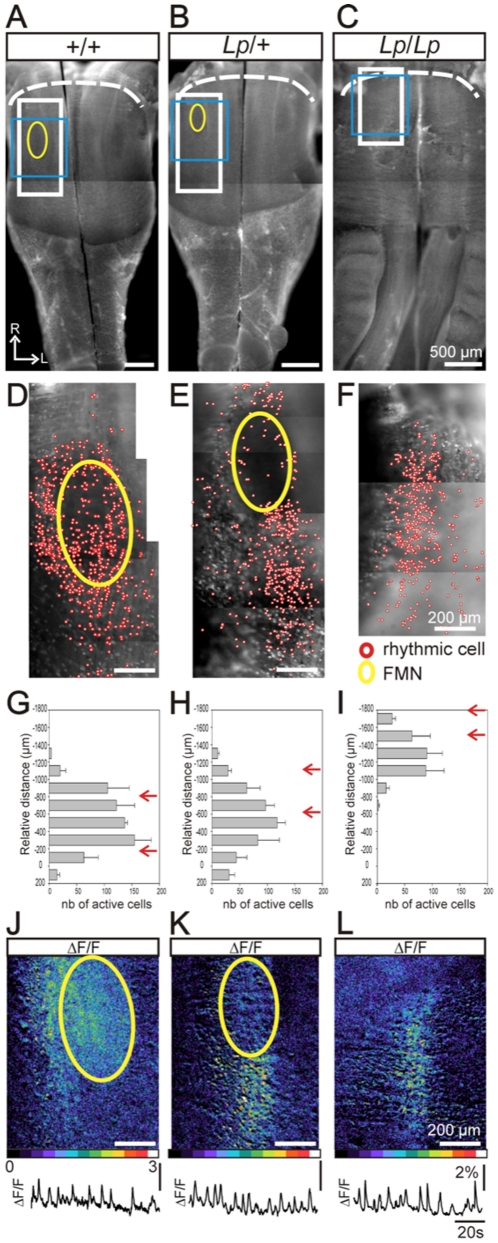
Altered distribution of active cells at the pial surface of *Lp* mutant hindbrain preparations. A–C: Ventral view of whole hindbrain preparations from E15.5 *+/+* (A), *Lp/+* (B) and *Lp/Lp* (C) embryos loaded with Calcium Green 1-AM and observed in direct fluorescence. Yellow ovals indicate the position of the facial motor nucleus (FMN) that is clearly visible in direct light (see right side of preparations in A and B). D–F: Maps for rhythmic active cells (red circles) detected at a high magnification for corresponding genotypes in the region delimited by the white rectangles in A–C. Active cells located at the ventral surface of the preparations are found for all genotypes. G–I: Histograms of the rostro-caudal distribution of active cells relative to the constant preBötC position for 2 wild-type (G), 3 *Lp/+* (H), and 3 *Lp/Lp* (I) embryos. The red arrows indicate the rostral and the caudal extremity of the FMN. The distribution of active cells shows a significant rostral displacement in *Lp/Lp* embryos. J–L: Calcium transients illustrated as relative fluorescent changes (ΔF/F) recorded in the region encompassing the active cells (delimited by the blue rectangles in A–C). Relative fluorescent changes intensity are color-coded, white corresponding to the strongest activity (see the color scale at the bottom). The traces below show the spontaneous calcium changes recorded over time in the entire active region for each genotype. R: rostral.

Since cells expressing e-pF markers are found in *Lp* mutants, we used calcium imaging to examine whether cells exhibiting the functional characteristics of e-pF neurons were present in *Lp* mutants. After loading isolated hindbrain preparations obtained from E15.5 *+/+*, *Lp/+* and *Lp/Lp* embryos with a calcium indicator (calcium green 1-AM), we searched for individual cells spontaneously generating rhythmically organized calcium transients (hereon referred to as active cells). Inspection of the entire ventral surface at high magnification revealed the presence of active cells for the three genotypes but in different locations. The FMN was visible in direct light on the ventral surface of +/+ and *Lp*/+ hindbrain preparations as a dark region (see right side of preparations in Fig. 3A, B). In *+/+* embryos (n = 3), individual active cells were located in the parafacial region: in a stripe flanking the lateral part of the FMN, and sparsely dispersed over and extending caudally to the FMN (Fig. 3A, D; cumulative distribution in Fig. 3G) as previously described for e-pF neurons [7]. In *Lp/+* embryos (n = 4), active cells were present in a large column starting from the caudal edge of the misplaced FMN, extending 300–400 microns more caudally and with a comparable medial position as the FMN (Fig. 3B, E; distribution in Fig. 3H). Few active cells were found lateral to the mis-located FMN. These data show that the rostro-caudal extension of the e-pF is preserved in *Lp/+* embryos despite a medial displacement. In *Lp/Lp* embryos (n = 3), although the structure of the neural tube was dramatically affected due to failure of neural tube closure, a significant number of active cells could be detected at the ventral surface of the hindbrain in a rostro-medial position (Fig. 3C, F; distribution in Fig. 3I). For all genotypes, the map of active cells (Fig. 3D, E and F), and their distribution along the rostro-caudal axis and relative to the FMN (Fig. 3G, H and I) coincides with the position of Phox2b^+^/Islet1,2^−^ expressing cells (Fig. 2). Together, these data suggest that individual cells sharing molecular or functional profiles with e-pF neurons are present at the ventral surface of the hindbrain in *looptail* mutants, even though they are displaced rostro-medially, concomitant with the failure of FBM neuron migration.

Next, we asked whether these active cells individually detected form together a functional network capable of generating a rhythmically organized activity. We examined, at low magnification, spontaneous population activities generated at the ventral surface of isolated E15.5 brainstem preparations, including those used to establish the maps of active cells. In *+/+* preparations (n = 10), rhythmic fluorescence changes, generated at a frequency of 10.4±0.5 burst/min, were detected laterally to the FMN (Fig. 3A), and were occasionally accompanied by a burst of activity in the FMN (Fig. 3J). In *Lp/+* preparations (n = 10), rhythmic calcium changes, occurring at a frequency of 12.1±0.8 burst/min, were observed caudal to the abnormally located FMN (Fig. 3B), in a region encompassing the location where Phox2b^+^/Islet1,2^−^ cells and individual active cells were detected (Fig. 3K), but not in a region immediately lateral to the mis-located FMN (the expected location for the e-pF relative to the FMN). In *Lp/Lp* preparations (n = 7), inspection of the entire ventral surface of the abnormally structured neural tube (Fig. 3C) revealed one active region located bilaterally at the rostral-most part of the preparation and in a medial position, corresponding to the area where e-pF-like neurons were found (Fig. 3L). In this region, rhythmic fluorescence changes were generated at a frequency of 10.5±0.9 burst/min. The activity of the FMN could not be detected because of its dorsal position. These data show that putative e-pF neurons in *Lp/+* and *Lp/Lp* embryos, despite being rostro-medially displaced, migrated normally to the ventral surface of the hindbrain to form a network that generates rhythmically organized spontaneous activity, suggesting the presence of a functional e-pF oscillator in these mutants.

### The active network present in *looptail* mutant hindbrains exhibits functional properties of the e-pF oscillator

**Figure 4:**
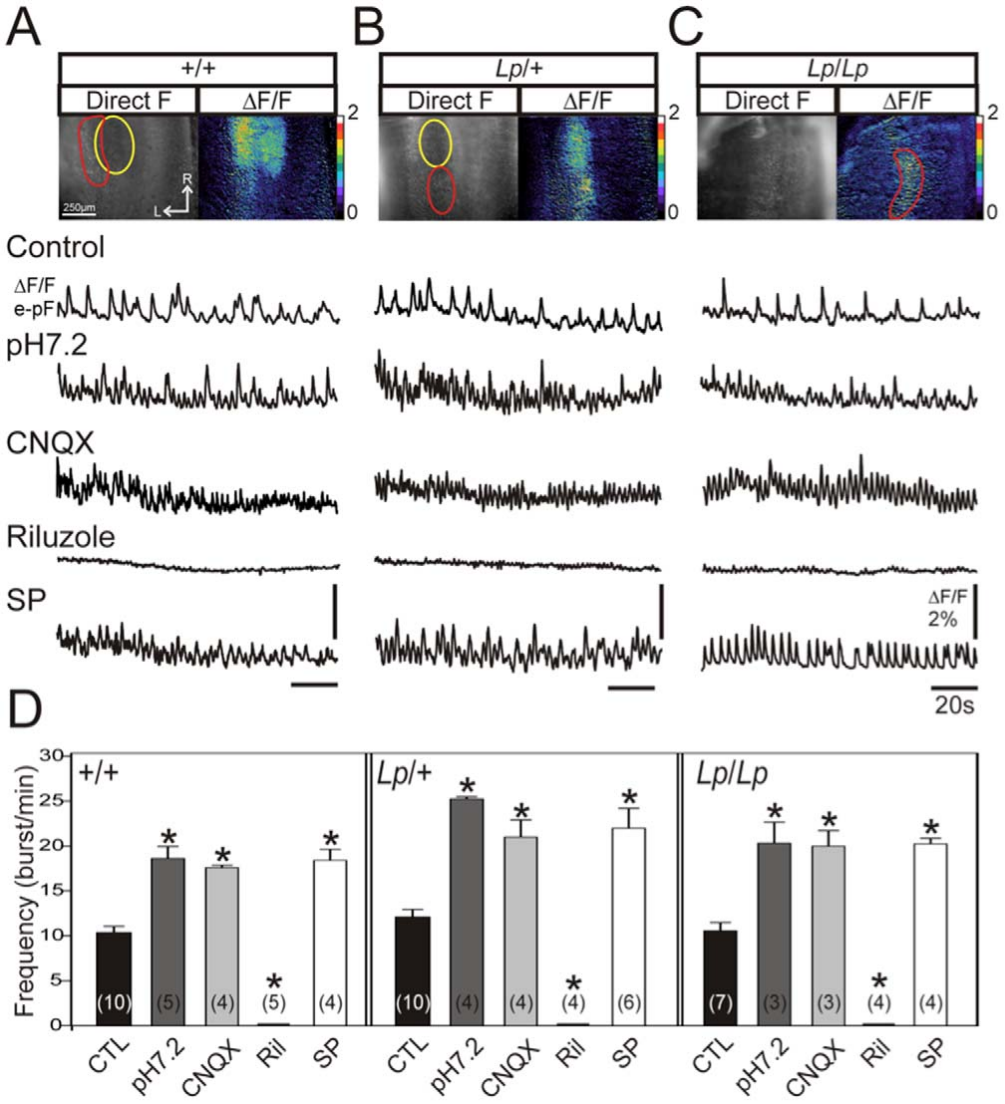
Active cells form a functional e-pF oscillator in *Lp* mutant embryos. A: Ventral view of the hindbrain over the area encompassing the FMN and the active region. Preparations were obtained from an E15.5 *+/+* embryo, loaded with calcium Green 1-AM and observed in direct fluorescence (left panel) or as relative changes in fluorescence (ΔF/F, right panel). The yellow oval indicates the position of the FMN, the e-pF network is encircled in red. Traces below indicate calcium transients (ΔF/F) measured in the e-pF region in control conditions (top trace), pH 7.2 (second trace), 10 µM CNQX (third trace), 10 µM Riluzole (fourth trace) and 10^−7^ M Substance P (SP, fifth trace). B and C: Same legend as in A for a *Lp/+* embryo (B) and a *Lp/Lp* embryo (C). D: Graphs representing the mean frequency of calcium transients measured in the e-pF region in different experimental conditions (indicated below and color coded) for *+/+* (left part), *Lp/+* (middle part) and *Lp/Lp* (right part). Numbers in brackets indicate the number of preparations tested. Asterisks indicate statistically different means. Frequency is increased in the presence of CNQX, SP and in pH 7.2 for all genotypes, while riluzole blocks the rhythmic activity in the active region. The active network found in the mutant shares pharmacological characteristics of the e-pF recorded in the wild type littermates. L: lateral, R: rostral.

Since active cells in *Lp/+* and *Lp/Lp* embryos are mis-located relative to +/+ embryos, it is possible that active neuronal groups other than those constituting the e-pF oscillator could have reached the ventral surface of the hindbrain and be the source of the rhythmic calcium variations observed in these preparations. Therefore, we characterized the active networks present in *Lp/+* and *Lp/Lp* embryos by testing their sensitivity to pharmacological agents known to change e-pF activity, including their response to acidosis [7]. First, application of an acidic bathing medium (pH 7.2) induced a significant increase in the frequency of the network activity for the three genotypes (Fig. 4, second traces in A, B and C; summarized in D). Second, blockade of glutamatergic transmission with 10 µM CNQX did not prevent rhythmic activity from being generated, and even induced an e-pF-characteristic frequency increase in wild-type [7], as well as in *Lp/+* and *Lp/Lp* embryos (Fig. 4, third traces in A, B and C; summarized in D). Third, blockade of the persistent sodium current with 10 µM riluzole completely blocked rhythmogenesis for all genotypes (Fig. 4, fourth traces in A, B and C; summarized in D). Finally, frequency activity significantly increased for all genotypes in the presence of 10^−7^ M Substance P, the preferred ligand for NK1R (Fig. 4, fifth traces in A, B and C; summarized in D), and consistent with the presence of NK1R-positive cells in this region (Fig. 1). These data indicate that the network generating rhythmic activity in the *Lp/+* and *Lp/Lp* embryos exhibited characteristics of the e-pF described in *+/+* preparations and in previous work [7]. Thus, despite a mis-location of the FMN, and a concomitant displacement of cells expressing e-pF markers, the e-pF oscillator forms and functions normally in *Lp/+* and *Lp/Lp* embryos.

**Figure 5:**
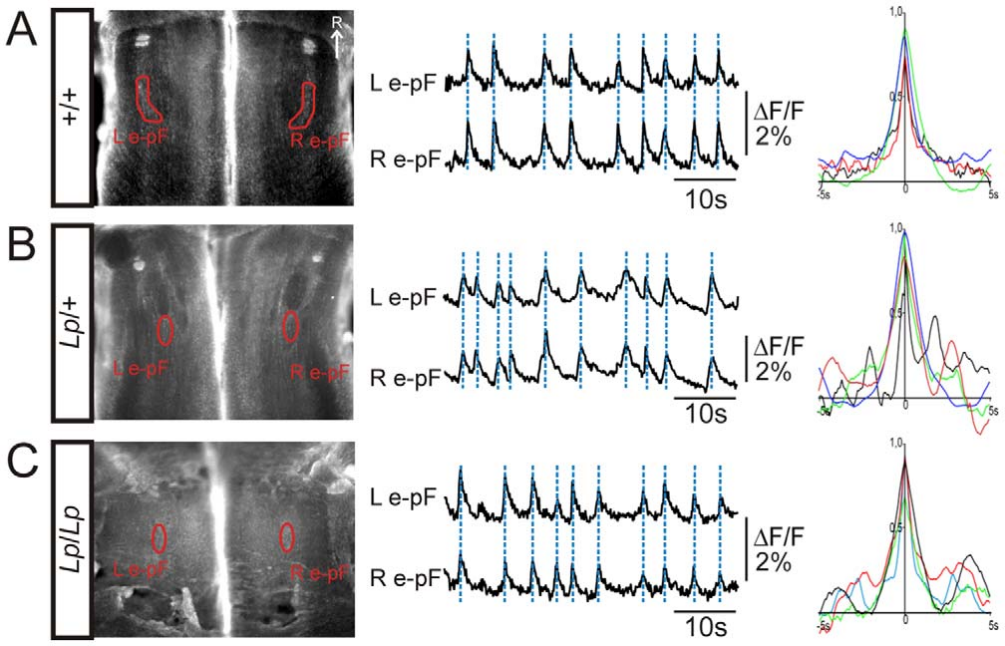
Bilateral e-pF oscillators exhibit synchronized activity in *Lp* mutant embryos. A–C: Left: Ventral view of whole hindbrain preparations from E15.5 *+/+* (A), *Lp/+* (B) and *Lp/Lp* (C) embryos loaded with Calcium Green 1-AM and observed in direct fluorescence. The red lines indicate the position of the left (L) and the right (R) e-pF oscillators. The two traces (middle panels) correspond to the calcium transients illustrated as relative fluorescent changes (ΔF/F) recorded simultaneously in the corresponding e-pF oscillators in one preparation. The dashed lines in blue highlight the synchronicity between the activities of both e-pF oscillators. Right: corresponding superimposed left-right e-pF cross-correlograms illustrated for 4 different preparations. Bilateral synchronous activity in the two e-pF oscillators is preserved the *Lp*/+ and *Lp*/*Lp* mutants. R: rostral.

The e-pF oscillators are bilaterally distributed in the hindbrain and functionally synchronized through poorly characterized commissural connections [7], [28]. We examined whether the displaced e-pF oscillators in *Lp* mutants were synchronously active by performing calcium imaging simultaneously in the two e-pF oscillators. In +/+ preparations, spontaneous fluorescent changes recorded at the ventral surface of hindbrain occurred simultaneously and synchronously in the two regions encompassing the e-pF oscillators (Fig. 5A). Similarly, in preparations obtained from *Lp*/+ (Fig. 5B) and *Lp*/*Lp* (Fig. 5C) embryos, rhythmically organized calcium transients were generated in phase in the two e-pF oscillators. Cross-correlograms in Figure 5 represent superimposed curves obtained from 4 different preparations for each genotype. In every preparation tested, rhythmic activity was generated synchronously in the left and the right e-pF oscillator. These data demonstrate that the bilateral synchrony of the e-pF oscillators is preserved in *Lp*/+ and *Lp*/*Lp* mutants, suggesting that commissural projections between the oscillators are established in the *Lp* mutants despite their abnormal positions.

### The location and activity of the preBötC oscillator are not affected in *looptail* mutants

**Figure 6:**
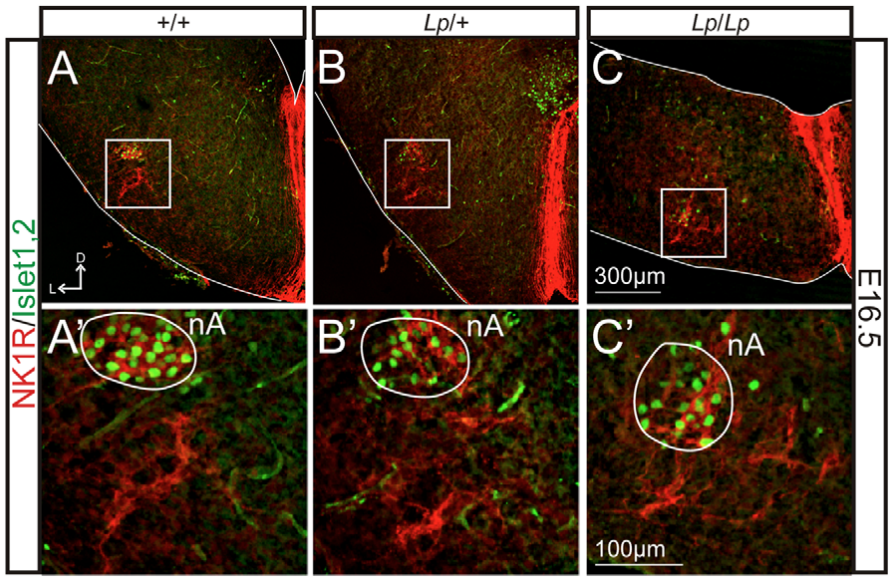
The preBötC oscillator is preserved in *Lp/Lp* mutants. Anti-NK1R (red) and anti-Islet1,2 (green) immunofluorescence on hindbrain transverse slices obtained from E16.5 embryos for +/+ (A, A′), *Lp/+* (B, B′) and *Lp/Lp* (C, C′) genotypes at the level of the preBötC area. The panels in A′–C′ are higher magnification views of the preBötC region delimited by white rectangles in A–C, respectively. The preBötC oscillator is defined as the NK1R-positive region located ventrally to the Islet1,2-positive nucleus ambiguous (nA). The preBötC is anatomically preserved in the *Lp/Lp* mutant. D: dorsal, L: lateral.

The second respiratory oscillator, the preBötzinger complex (preBötC) is adjacent to motor neurons composing the nucleus ambiguus (nA). While the *looptail* mutation affects the position of the FMN [16], its effects on the position of the nA motor neurons and the adjacent preBötC interneurons have not been examined. We addressed this issue by confirming the anatomical data presented in Figure 2 using the position of the nA as a landmark to check for the expression of a preBötC marker (NK1R), combined with a functional analysis examining the generation of rhythmic activity in the preBötC area. E16.5 hindbrains were transversally sectioned and processed for immunostaining with anti-NK1R antibody (preBötC and nA marker) and Islet1,2 antibody to label the adjacent motor neurons of the nA. In *+/+* embryos (n = 3; see also [8], [28]), NK1R-positive cells were located ventral to the Islet1,2-positive cells of the nA, starting 250–300 µm caudal to the posterior limit of the FMN (defining the “r6” location), and extending caudally over 400 µm (Fig. 6A, A′; [28]. In *Lp/+* embryos (n = 3), the NK1R- positive cells started 500–600 µm caudal to the posterior limit of the FMN (defining the “r5” location), and extended caudally over 400 µm (Fig. 6B, B′). In *Lp/Lp* embryos (n = 2), NK1R-positive cells were located ventral to the nA, starting 1200–1400 µm caudal to the posterior limit of the FMN (defining the “r4” location), and extending caudally over 300 µm (Fig. 6C, C′).

**Figure 7:**
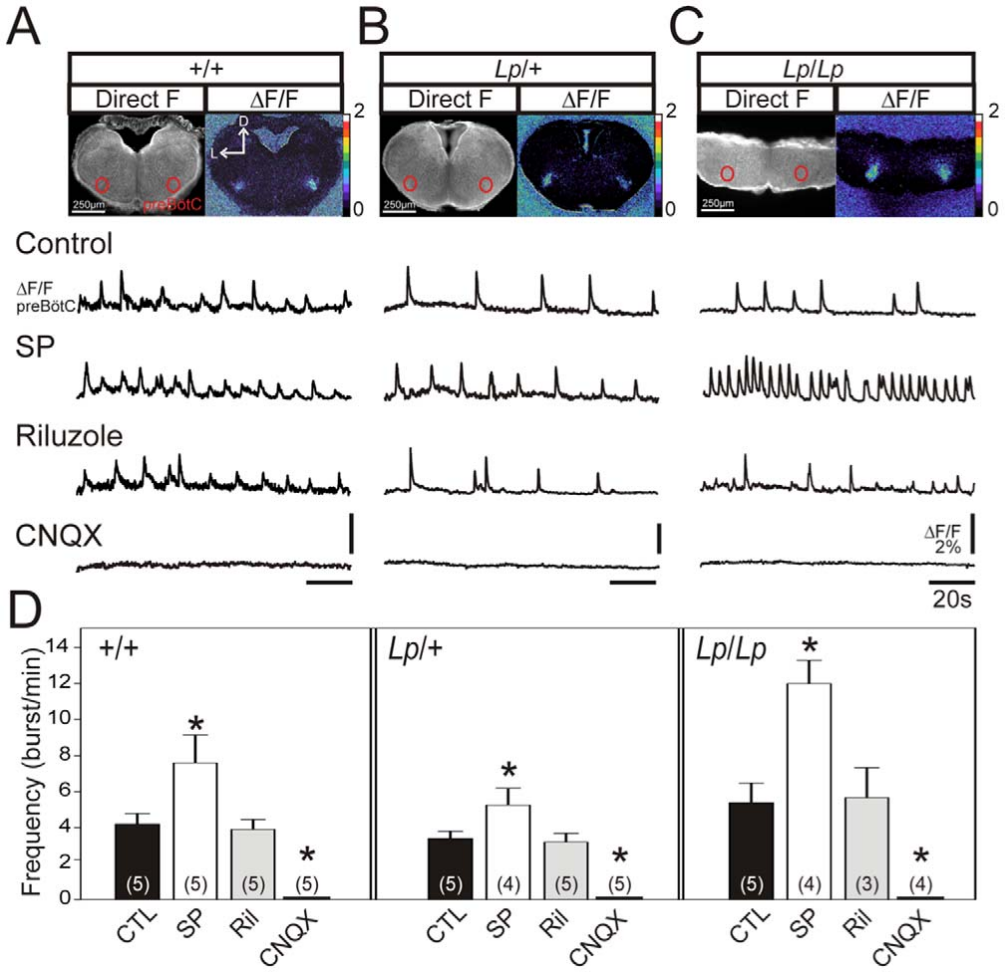
Functional analysis of the preBötC oscillator in the *Lp* mutant. A: Photomicrograph of a transverse medullary slice through the preBötC obtained from an E16.5 *+/+* embryo loaded with calcium green 1-AM observed in direct fluorescence (left panel) and as relative changes in fluorescence (ΔF/F, right panel). The red circles indicate the position of the bilaterally distributed preBötC oscillators. Traces below (ΔF/F preBötC) indicate calcium transients measured in the preBötC region in control conditions (top trace), 10^−7^ M Substance P (SP, second trace), 10 µM Riluzole (third trace) and 10 µM CNQX (fourth trace). B and C: Same legend as in A for a *Lp/+* embryo (B) and a *Lp/Lp* embryo (C). D: Graph representing the mean frequency of calcium transients measured in the preBötC region in different experimental conditions (indicated below and color coded) for *+/+* (left part), *Lp/+* (middle part) and *Lp/Lp* (right part). Numbers in brackets indicate the number of preparations tested. Asterisks indicate statistically different means. Frequency is increased in the presence of SP, unchanged in the presence of riluzole and blocked in the presence of CNQX for all genotypes. Hence, the preBötC oscillator is functionally preserved in *Lp/Lp* mutants. D: dorsal, L: lateral.

Based on these anatomical data, transverse medullary slices were prepared from control and *looptail* mutant embryos, and preBötC rhythmic activity was recorded using calcium imaging. Spontaneous and rhythmically organized calcium transients were present in all slice preparations examined irrespective of the genotype (Fig. 7A, B, C, first traces). Activity frequencies did not differ significantly among genotypes (summarized in Fig. 7D). Mean frequency was 4.2±0.5 burst/min in +/+ slices, 3.4±0.4 burst/min in *Lp/+* slices and 5.4±1 burst/min in *Lp/Lp* slices. Exogenous application of SP (10^−7^ M) induced a significant frequency increase (∼1.5–2.2 fold) over baseline in all three genotypes (Fig. 7A, B, C, second traces; summarized in Fig. 7D). In contrast, 10 µM riluzole did not change activity frequency, while 10 µM CNQX blocked rhythm generation in all preparations and all genotypes (Fig. 7A, B, C, two bottom traces; summarized in Fig. 7D). These results demonstrate that rhythmically active preBötC oscillators are present in *looptail* mutants. Taken together with the immunostaining analysis, these data indicate that the preBötC oscillator develops in the correct location and functions normally in *Lp/+* and *Lp/Lp* embryos.

### Functional coupling between the e-pF and preBötC oscillators in *Lp/+* embryos

**Figure 8:**
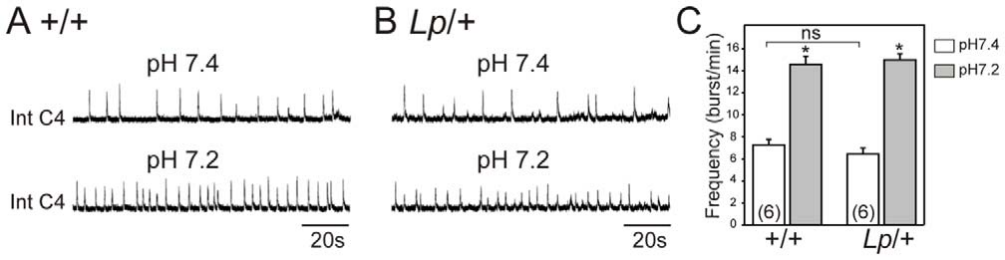
Response to a low pH challenge is unaffected in the *Lp/+* mutant. Integrated phrenic nerve discharge (Int C4) at pH 7.4 (upper trace) and pH 7.2 (bottom traces) for control (A) and *Lp*/+ (B) embryos at E16.5. C: Quantification of burst frequencies for control (left) and heterozygous (right) embryos at pH 7.4 (white bars) and pH 7.2 (gray bars). Numbers of hindbrain preparations analyzed are indicated on the bars. The motor output of the respiratory network recorded from C4 nerve roots is comparable in *Lp/+* and wild type preparations in control conditions, and the response to low pH is preserved in the *Lp/+* mutant.

Finally, we investigated whether a functional coupling between the two respiratory oscillators was established in *Lp* mutants. Hence we examined the consequences of a change in the pH of the bathing solution on the activity of the preBötC through recording of the phrenic nerve (C4) root activity. We showed previously that the e-pF is intrinsically sensitive to acidification [7], whereas the preBötC is not [9], and that the two oscillators are functionally coupled at E16.5 [7]. In control embryos (n = 6), nerve recordings from C4 roots revealed stable rhythmic bursting activity generated at a mean frequency of 7.3±0.5 burst/min. Lowering the pH from 7.4 to 7.2 induced a significant increase in C4 activity frequency up to 14.6±0.7 burst/min (Fig. 8A, C). Similarly, in preparations obtained from *Lp*/+ embryos (n = 6), rhythmic phrenic discharges, generated at a frequency of 6.5±0.5 burst/min at pH 7.4, increased in frequency to 14.9±0.6 burst/min at pH 7.2 (Fig. 8B, C). These results show that pH sensitivity is preserved in the *Lp*/+ mutant. In addition, there was no statistical difference in C4 frequencies between the two genotypes in control conditions (pH 7.4), suggesting that the preBötC receives an excitatory input from the displaced e-pF in *Lp/+* heterozygotes. Together, these data strongly suggest that the e-pF and the preBötC oscillators are functionally coupled, despite the abnormal position of the e-pF in the *Lp*/+ mutants. We could not examine functional coupling between the oscillators in *Lp*/*Lp* mutant embryos due to the absence of phrenic roots.

## Discussion

**Figure 9:**
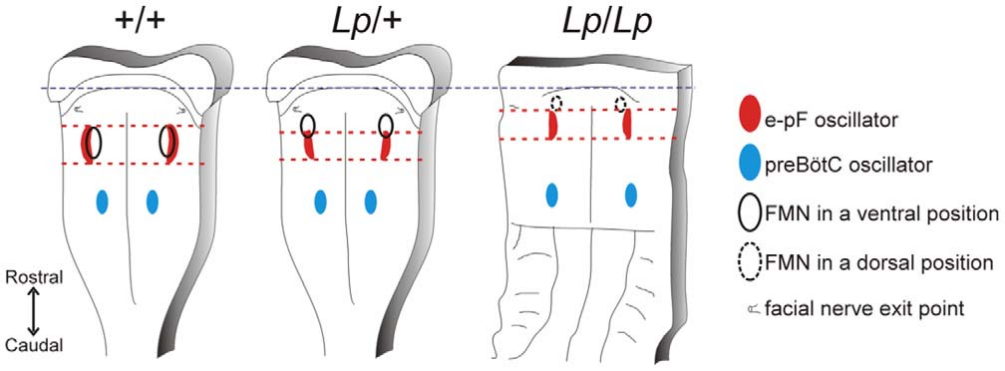
Schematic summary of the relative positions of respiratory oscillators and the FMN in the hindbrain in *+/+*, *Lp/+* and *Lp/Lp* embryos. Hindbrain outlines were drawn using photomicrographs of isolated preparations as a template. The dashed blue line indicates the landmark used to align the preparations (position of the caudal limit of migrating pontine neurons). The red dashed lines indicate the rostral and caudal limits of the e-pF oscillator (red ovals). The blue ovals indicate the preBötzinger complex. The position of the facial motor nucleus (FMN) is indicated by the black ovals, shown with a dashed line in the *Lp/Lp* embryo due to its dorsal position. The e-pF oscillator is slightly displaced rostro-medially in *Lp/+* embryos, and significantly displaced rostrally in *Lp/Lp* embryos relative to +/+ embryos.

Using calcium imaging and immunostaining on isolated hindbrain preparations from the Wnt/PCP *Vangl2* mutant *looptail*, we show here that neurons constituting the embryonic parafacial and preBötC respiratory oscillators are specified correctly and form functional oscillators generating rhythmically organized activities even though the e-pF is mis-positioned in mutant embryos (Fig. 9). Significantly, in *Lp*/+ mutants, chemosensitive entrainment of the respiratory-like rhythm is conserved, indicating that inter-oscillator connectivity may also be preserved. These data indicate that robust developmental processes are involved in the establishment of the two distinct respiratory oscillators required for breathing regardless of a largely disturbed hindbrain anatomy.

### Failure of e-pF neuron caudal migration in *Lp/Lp* embryos

In *Lp/+* embryos, the e-pF neurons were displaced medially, but their rostro-caudal positions (defined by activity mapping and immunostaining) were less affected (Fig. 9). In addition, the preBötC could be definitively identified in *Lp/+* embryos based on the position of the nA. By contrast, the e-pF and preBötC neurons (defined by activity mapping and pharmacology) were farther apart in *Lp/Lp* embryos compared to *Lp/+* embryos (Fig. 3), consistent with a significant rostral displacement of the e-pF neurons. Given that the dimensions of the rhombomeres and their patterning are not affected in *Lp/Lp* embryos (Fig. 1), and that the rostro-caudal position of the preBötC is similar between *Lp/Lp* and *Lp/+* embryos (Fig. 2), the location of the FBM neurons in r4 in *Lp/Lp* embryos indicates that e-pF neurons in these embryos are mostly located in r5 rather than r6 (Fig. 9), reflecting in these neurons a failure of caudal migration out of r5.

### Establishment of functional respiratory oscillators in *looptail* mutants

Proper development of and interconnection between the e-pF and preBötC oscillators is essential for functional breathing. We provide several lines of evidence for an effective coupling between the e-pF and the preBötC in *looptail* mutants. First, one function of the e-pF is to increase respiratory frequency through interconnections with the preBötC. This rhythm-promoting function of the e-pF was preserved in *Lp/+* embryos, since the preBötC frequency was significantly slower in slices (lacking the e-pF) than in whole hindbrain preparations (including the e-pF). Second, blockade of glutamatergic connections with exogenous application of CNQX induced in all genotypes, an increase in e-pF activity frequency, likely resulting from the silencing of the preBötC oscillator [7], and freeing the e-pF from the frequency constraint imposed by interconnections with the preBötC. Third, the increased phrenic frequency in response to acidosis in *Lp/+* mutants also indicates that the two respiratory oscillators are functionally connected. In addition, we have shown that the two e-pF oscillators are bilaterally synchronized. This demonstrates that commissural connections between the two e-pF oscillators are established both in *Lp*/+ and *Lp*/*Lp* mutants. Taken together, multiple aspects of the circuitry underlying the function of the respiratory rhythm generator are maintained in the *looptail* mutants regardless the altered anatomical position of one of its two key components.

Another function of the e-pF/pFRG is to mediate the central response to acidosis, even at embryonic stages [9], [29], [30]. Gourine et al. (2010) [11] identified astrocytes as an important component for chemoreception in adults, since they are associated with blood vessels at the ventral surface of the hindbrain, and have the ability to modulate breathing pattern by responding to pH changes. The ventral position of e-pF neurons in the embryo may be a pre-requisite for a functional interaction with these astrocytes. Our observation that a change in the pH of the bathing medium induces a change in the frequency of e-pF activity in *Lp*/+ and *Lp/Lp* embryos, and is able to affect phrenic nerve activity in *Lp*/+ embryos, indicates that the neuronal response to acidosis is preserved in *Lp* mutants. Thus, although many e-pF neurons fail to move rostro-caudally out of r5 location, they migrate normally toward the ventral surface of the brainstem, enabling interaction with the astroglial component of the central chemoreception mechanisms.

### Mechanisms regulating e-pF neuron migration

The radial migratory pathways of the FBM and e-pF neurons overlap in space and time. Following caudal migration from r4 into r6 between E11.5–13.5, FBM neurons migrate radially toward the pial surface to form the FMN [25], [31], [32]. Similarly, after caudal migration into r6 between E12–14, e-pF neurons migrate radially past the FMN to form the e-pF at the pial surface, ventral to the FMN [10]. Since the closely-apposed FBM and e-pF neurons take similar migratory pathways toward the ventral surface in r6, it is possible that 1) the e-pF neurons are dependent on FBM neurons for migratory cues, or 2) the e-pF and FBM neurons migrate independently of each other using common or distinct environmental cues. The latter possibility is reasonable, given that both e-pF and FBM neurons express *Phox2b*, which could regulate expression of migration-associated genes. However, numerous studies now indicate that e-pF and FBM neurons migrate independently of each other. First, in *Egr2* knockout and *Phox2b*-conditional mutant embryos, FBM neurons migrate normally and form the FMN even though the e-pF neurons are anatomically and functionally absent [7], [9], [20]. Importantly, e-pF neurons migrate normally in the complete absence of FBM neurons in *Islet1^cre/+^*; *Phox2b^flox/flox^* mutants [9], although their ability to constitute a functional oscillator remains to be established in this context. Consistent with these observations, our data show that irrespective of the position of the FMN (ventral, rostral or even dorsal) in *Lp/+* and *Lp/Lp* embryos, e-pF neurons migrate radially in a normal fashion to the ventral surface in the r5-r6 region, and form an active network generating rhythmically organized activity. Interestingly, many e-pF neurons are located rostrally, in an r5 position, in *Lp/Lp* mutants. This mis-location may result from failure of the neurons to migrate caudally out of their putative birthplace in r5. Since this failure to migrate mirrors the failure of FBM neurons to migrate out of r4 in *Lp/Lp* mutants, *Vangl2* may be independently required for the caudal migration of FBM neurons and e-pF neurons. *Vangl2* is likely to regulate these neuronal migrations by functioning in other cell types since the gene functions non cell-autonomously to regulate facial motor neuron migration in zebrafish [13], and is not expressed in e-pF neurons at all stages tested (E12.5–14.5), spanning their migratory phase (DMG and AC, unpublished data). Unlike FBM neurons, which migrate to form a compact nucleus (FMN), e-pF neurons typically show a broad rostro-caudal distribution such that significant numbers of e-pF neurons are found hundreds of microns caudal to the FMN. As a result, even in *Lp/Lp* embryos, some e-pF neurons are located in the r6 region (Fig. 2), even though most of them fail to migrate out of the r5 region. This suggests that other mechanisms, in addition to *Vangl2*-mediated signaling, may also regulate e-pF neuron migration.

### Consequences of defective neuronal positioning on physiological function

The link between defective neuronal patterning and altered physiology is supported by studies of mouse cerebellum mutants where neural circuits regulating motor coordination are affected [33], and of hindbrain segmentation mutants where neural networks controlling respiration are defective [7], [34]. Therefore, it is notable that the e-pF and preBötC oscillators exhibit normal physiological characteristics in *Lp/+* and *Lp/Lp* embryos despite mis-positioning of e-pF neurons.

*Lp/+* embryos survive to adulthood and reproduce, indicating that breathing and jaw-associated chewing behaviors are functional even though the e-pF neurons and mainly the FBM neurons, which respectively control these behaviors, are mis-positioned in these animals. These observations demonstrate that neural circuits can tolerate changes in the positioning of component neurons. The bilaterally located e-pF neurons are coupled to each other through poorly characterized commissural connections [7]. However, inspite of being mis-positioned in *Lp* mutants, e-pF oscillators remain bilaterally synchronized, indicating that left/right commissural connectivity within the respiratory rhythm generator is preserved. Therefore, the neuroanatomy and functionality of neuronal groups in the hindbrain, especially of the FBM neurons and e-pF neurons that we have studied, may be resistant to changes in neuronal position resulting from defective migration.

In conclusion, our findings show that the specification, connectivity and activity of the e-pF respiratory oscillator are largely insensitive to neuronal migration events mediated by the Wnt/PCP component Vangl2. Additional experiments are required to define the guidance mechanisms critical for establishing the neural network that regulates breathing. Importantly, we show that a functional respiratory circuit can be established inspite of profound neuronal migration and neural tube closure defects in the hindbrain, thus highlighting the robustness of developmental events participating in the formation of neuronal oscillators mediating an essential physiological function.
